# Timing, optimal dose and intake duration of dietary supplements with evidence-based use in sports nutrition

**DOI:** 10.20463/jenb.2016.0031

**Published:** 2016-12-31

**Authors:** Alireza Naderi, Erick P. de Oliveira, Tim N. Ziegenfuss, Mark E.T. Willems

**Affiliations:** 1 Department of Sport Physiology, Boroujerd Branch, Islamic Azad University, Boroujerd Iran; 2 School of Medicine, Federal University of Uberlandia, Uberlandia, Minas Gerais State Brazil; 3 The Center for Applied Health Sciences, Stow, Ohio USA; 4 Department of Sport and Exercise Sciences, University of Chichester, College Lane, Chichester United Kingdom

**Keywords:** Sports nutrition, Dietary supplements, Timing, Dose, Intake duration

## Abstract

**[Purpose]:**

The aim of the present narrative review was to consider the evidence on the timing, optimal dose and intake duration of the main dietary supplements in sports nutrition, i.e. β-alanine, nitrate, caffeine, creatine, sodium bicarbonate, carbohydrate and protein.

**[Methods]:**

This review article focuses on timing, optimal dose and intake duration of main dietary supplements in sports nutrition.

**[Results]:**

This paper reviewed the evidence to determine the optimal time, efficacy doses and intake duration for sports supplements verified by scientific evidence that report a performance enhancing effect in both situation of laboratory and training settings.

**[Conclusion]:**

Consumption of the supplements are usually suggested into 5 specific times, such as pre-exercise (nitrate, caffeine, sodium bicarbonate, carbohydrate and protein), during exercise (carbohydrate), post-exercise (creatine, carbohydrate, protein), meal time (β-alanine, creatine, sodium bicarbonate, nitrate, carbohydrate and protein), and before sleep (protein). In addition, the recommended dosing protocol for the supplements nitrate and β-alanine are fixed amounts irrespective of body weight, while dosing protocol for sodium bicarbonate, caffeine and creatine supplements are related to corrected body weight (mg/kg bw). Also, intake duration is suggested for creatine and β-alanine, being effective in chronic daily time < 2 weeks while caffeine, sodium bicarbonate are effective in acute daily time (1-3 hours). Plus, ingestion of nitrate supplement is required in both chronic daily time < 28 days and acute daily time (2- 2.5 h) prior exercise.

## INTRODUCTION

An increased health awareness among athletes and the public will favor the global sports nutrition market which is predicted to have an annual growth rate of 9% from 2013 to 2019, to an estimated value of USD 37.7 billion in 2019 (http: //www.transparencymarketresearch. com/sports-nutrition-market.html). High use of dietary and nutritional supplements has been reported in various regions. For example, supplement intake by elite Finnish athletes was 81% in 2002 and 73% in 2009^1^. In Iran, women and men body builders reported use of 11% and 87%, respectively^2^. Athletes use dietary supplements to: 1. aid recovery from training; 2. acquire health benefits; 3. treat illness; and/ or 4. compensate for a poor diet^3^. According to the US Food and Drug Administration, a dietary supplement is a product (other than tobacco) to supplement the diet and contains one or more of the following ingredients; a vitamin, a mineral, an amino acid, a herb or other botanical. It supplements the diet by increasing its total daily intake, or a concentrate, metabolite, constituent, extract, or combination of these ingredients^4^. Many sport supplements are available, however, it is only a few that are supported by enough evidence to have a performance effect including β-alanine, beetroot juice (nitrate), caffeine, creatine, sodium bicarbonate, carbohydrate and protein^5^. Additionally, studies have also addressed supplementation and nutrient timing. Supplementation timing can be described as the strategy for intake at a specific time. For example, muscle protein synthesis (MPS) may be higher with protein ingestion after resistance exercise compared to intake before exercise^6^. In addition, creatine monohydrate immediately after body building exercise provided greater improvement of fat-free mass and body composition compared to intake before exercise^7^. However, there is still no consensus for the timing for these supplements. In addition to supplementation timing, the optimal dosage also needs to be considered. For example, a dose of 20 g of post-exercise protein did further increase mTOR signaling in skeletal muscle compared to 10 g^8^, and it was observed that a dose higher than 300 mg/kg of sodium bicarbonate likely causes gastrointestinal discomfort^9^.

This narrative review focuses on timing and optimal dosage and intake duration of the main sport supplements related recently in the new American College of Sports Medicine position^5^. Two categories of supplements were considered, i.e. the acute daily-used supplements: beetroot juice (nitrate), caffeine and sodium bicarbonate and the chronic daily-used supplements: β-alanine, creatine, carbohydrate, and protein.

Literature was retrieved based on a search in Pubmed (1990-2016) and Google Scholar (1990-2016). Search engines used the keywords combined: “creatine timing”, “creatine exercise”, “creatine supplementation”, “beetroot juice supplementation”, “beetroot juice dose”, “caffeine”, “caffeine timing”, “caffeine exercise”, “bicarbonate timing”, “sodium bicarbonate exercise”, “beta-alanine timing”, “beta-alanine exercise”, “beta-alanine dose”, “protein exercise”, “protein timing”, ”carbohydrate exercise”, “carbohydrate supplementation”. The literature search was limited to randomized controlled trials with humans. Articles related to supplements, linked with timing, dosage and duration in response to exercise were considered. References in retrieved articles were considered. Our current state of knowledge is that dose-response studies, analysis of blood and tissue parameters, and performance analysis studies allowed the establishment of guidelines of effective dosing strategies for the sports nutrition supplements nitrate, caffeine, sodium bicarbonate, β-alanine, creatine, carbohydrate, and protein.

## Nitrate-rich Beetroot Juice and Metabolism of Nitric Oxide

Nitric oxide is a labile soluble signaling molecule, synthesized from L-arginine by nitric oxide synthase that can modulate skeletal muscle contractility, glucose homeostasis and mitochondrial respiration^10^. Nitric oxide bioavailability is enhanced by intake of nitrate-rich beetroot juice by alteration of nitrate to nitrite and conversion to nitric oxide^11^^, ^^12^. This may be more important in hypoxic situations, particularly during exercise^13^. Moreover, beetroot juice has beneficial effects on submaximal exercise lasting 5-30 min^14^.

### Optimal Dosage, Timing and Duration of Nitrate- rich Beetroot Juice

Nitrate-rich beetroot containing 5-7 mmol of nitrate reduces resting blood pressure and the oxygen cost of sub-maximal exercise and improves exercise performance^15^. Wylie et al^16^ reported in a dose-response study that acute ingestion of beetroot (containing 70 mL, 140 mL or 280 mL nitrate) increased nitrate and nitrite in plasma, 5, 8 and 18 fold, respectively. However, only doses of 140 mL (8.4 mmol) and 280 mL (16.8 mmol) reduced the oxygen cost during moderate-intensity cycling and improved the time to exhaustion during severe-intensity cycling compared to placebo. No additional improvement was observed in exercise performance going from a dosage of 140 mL (8.4 mmol) to 280 mL (16.8 mmol). In agreement with this finding, Hoon et al^17^ observed better performance in a 2,000 m rowing ergometer time trail with nitrate-rich beetroot juice containing 140 mL (8.4 mmol) nitrate within 2 h prior to the test compared with 70 mL (4.2 mmol). More recently, Jones^14^ stated that 5-9 mmol nitrate/day during 1-15 days seems to have beneficial effects on the physiological responses to exercise. In addition, Wylie et al^18^ examined the chronic effect of beetroot supplementation at dosages of 3 mmol and 6 mmol on the O_2_ cost of submaximal exercise with and without acute ingestion of beetroot (2 h prior exercise). The results showed that despite a significant elevation in plasma [NO_3_^-^] and [NO_2_^-^] throughout the supplementation, neither 7 days nor ~4 weeks of low dose (3 mmol) beetroot supplementation reduce the O2 cost during submaximal cycle exercise, In contrast, the higher elevation in plasma [NO_3_^-^] and [NO_2_^-^] following 6 mmol beetroot supplementation was along with a reduction in the O2 cost of submaximal cycle exercise after 2 h (P = 0.06), 7 days and ~4 weeks (both P < 0.05) of supplementation. The study also showed that the reduction in submaximal exercise VO_2_ after ~4 weeks supplementation with 6 mmol NO_3_^-^ is preserved up to 24 h after the latest dose of NO_3_^-^ is ingested and thus, in the absence of elevated plasma [NO_2_^-^]. Thus, it appears that nitrate-rich beetroot juice with 5-9 mmol of nitrate and ingested 2-2.5 hours prior to exercise for 1-28 days may improve performance^14^^-^^18^. A week of nitrate supplementation did not affect kidney function at rest and during submaximal exercise^19^.

## Caffeine

Caffeine (1,3,7-trimethylxanthine) is a naturally occurring psychoactive stimulant. Approximately 90% of adults, and up to 70% of competitive athletes use caffeine regularly^20^. Upon ingestion, caffeine is first metabolized by enzyme cytochrome P450 1A2 (CYP1A2) into theophylline (~84%), theobromine (~12%) and paraxanthine (~4%)^21^. Most of this first stage metabolism occurs in the liver, but brain and kidneys are also thought to play a role. Further metabolism results in formation of dimethyl- and monomethylxanthines, dimethyl and monomethyl uric acids, trimethyl- and dimethylallantoin, and uracil derivatives^21^. Caffeine by oral and IV administration shows similar pharmacokinetics with caffeine readily able to cross all biological membranes^22^. Worldwide, daily caffeine consumption is estimated to range from ~70 mg to more than 400 mg per day^23^. Oral ingestion of caffeine is rapidly absorbed into the bloodstream and peaks within 30-60 minutes^24^. Caffeine in caffeinated gums is absorbed through the buccal mucosa, and peak levels are achieved more rapidly^25^. Caffeine elimination varies widely due to genetic differences in cytochrome P450 1A2, but with doses of ~3-6 mg/kg bodyweight, half-life is generally 2.5-10 hours^26^. In addition, genetic polymorphisms in cytochrome P450 1A2 impact on performance improvements by caffeine, as AA homozygotes outperform C allele carriers, at least during endurance exercise^27^. Caffeine has effects on the central nervous system, mainly resulting from the antagonism of adenosine receptors^28^ with physiological responses that reduce pain perception, sustain attention and vigilance, increase alertness and enhance mood. Other mechanisms that may play a role in improving exercise performance include increased sodium/potassium pump activity (which may help to maintain the membrane potential), increased calcium release from the sarcoplasmic reticulum (which may enhance excitation-contraction coupling), inhibition of phosphodiesterase enzymes (which may increase cellular concentrations of cAMP and cGMP), and increased fat oxidation/glycogen sparing (thought to be active only during the initial 15-30 minutes of exercise). For a thorough review, the reader is referred to papers by Jones^26^, Davis and Gren^29^ and Meeusen et al^30^.

### Timing and Dose of Caffeine Ingestion

Caffeine intake of ~3-6 mg/kg body weight, when ingested 30-60 minutes pre-exercise, increased time-to-exhaustion, promoted greater work capacity, and reduced effort perception during endurance exercise^31^. Also, Spriet^32^ observed lower caffeine intake (≤ 3 mg/kg body mass) before exercise able to enhance exercise performance. Low caffeine intake improved cognitive performance such as vigilance, alertness and mood during and following exhaustive exercise through its effect on the central nervous system. In addition, pre-exercise caffeine ingestion improved high-intensity activities lasting 4-180 seconds^29^ and performance in a variety of team sports^31^. Higher doses of caffeine do not provide greater benefits and are associated with side effects. Caffeine effects on strength and power performance is equivocal with some studies reporting benefits^33^^, ^^34^ while others do not^36^^-^^37^. Chronic caffeine ingestion upregulates CYP1A2, speeds metabolic clearance, and results in habitation in most, but not all users^37^. As such, abstention from caffeinated foods and beverages for a few days prior to an event may maximize performance effects. Recently, however, morning ingestion of caffeine improved neuromuscular function (contraction velocity) and 3-km time trail cycling performance^38^^, ^^39^ with lower negative side-effects compared to e vening ingestion of caffeine^38^. The positive effect of caffeine was attributed to higher activity of CYP1A2 in the morning than evening^39^.

### Sodium Bicarbonate

Bicarbonate is an extracellular buffer regulating blood pH. In a physiological environment, when a strong acid is added to extracellular fluid, bicarbonate ions act as a weak base reducing the hydrogen released by the stronger acid and forming carbonic acid^40^. Large amounts of bicarbonate ingestion before exercise enhance the bicarbonate levels and pH capacity^41^. In addition, bicarbonate loading also benefits exercise performance in high intensity exercises lasting 1 to 7 min^41^. Although consistency in blood parameters with repeated bicarbonate intake was observed, the repeatability of the performance effect is ambiguous and single trials on performance effects should be treated with caution^42^.

### Timing and Dose of Sodium Bicarbonate Ingestion

To avoid or minimize gastrointestinal distress and improve exercise performance, the optimum dose of sodium bicarbonate needs to be known. McNaughton^9^ examined effects of sodium bicarbonate dose (i.e. 200, 300, 400 and 500 mg/kg) on maximal 60s cycling. Highest peak power and total work done were achieved with 300 mg/ kg sodium bicarbonate with no additional benefits with higher dosages. In line, Siegler et a^43^ observed that 200 and 300 mg/kg sodium bicarbonate further increased blood buffering capacity compared to 100 mg/kg. The peak of bicarbonate concentration was achieved 60 min after ingestion of 300 mg/kg sodium bicarbonate while most efficient time to consume 200 mg/kg sodium bicarbonate was 40-50 min prior to exercise. Also, the bicarbonate concentration reached a plateau after 90 min. Recently, Siegler et al^44^ examined the timing of bicarbonate intake (i.e. 60, 120 and 180 min) prior to repeated bouts of high intensity exercise. No differences were found in blood buffering capacity between 60-180 min post ingestion of sodium bicarbonate 300 mg/kg. However, less gastrointestinal discomfort was observed at 180 min post ingestion of sodium bicarbonate. In line, more recently, Stannard et al^45^ revealed a high-individual variability of peak blood bicarbonate with time ranging from 30-180 min following 0.1-0.3 g/kg BM of NaHCO_3_ supplementation. The authors suggested that the athletes need to individualise ingestion of NaHCO_3_ and trial varying doses prior to competition. They also advised that for those athletes needing to consume NaHCO_3_ ≤ 30 min prior onset of exercise, smaller doses could be ingested without side effects. In a meta-analysis by Carr et al^46^, it was reported that sodium bicarbonate at 300 mg/kg improved high intensity performance lasting 1 minute and a higher dosage (≥ 300 mg/kg bw) was required for repeated sprints. Stomach pain and diarrhea are major side effects^44^^-^^47^.

However, intake of several small doses along with intake of a meal containing carbohydrate and some fluid is recommended to reduce gastrointestinal discomfort of sodium bicarbonate intake^43^^, ^^48^. Thus, co-ingestion of sodium bicarbonate (300-500 mg/kg bw), 60-180 min prior to exercise along with a meal containing carbohydrate and some fluid minimized gastrointestinal symptoms and improved exercise performance.

### β-alanine

β-alanine is a non-proteogenic amino acid in foods such as fish and meat, and is a precursor and rate-limiting for carnosine synthesis^49^. Carnosine (i.e. β-alanyl-L-hystidine) is abundantly present in skeletal muscle with a concentration of ~5–8 mmol/L wet muscle or ~20–30 mmol/ kg dry muscle^50^. Carnosine is involved in intramuscular pH regulation, sarcoplasmic reticulum calcium regulation, enzyme regulation and antioxidant activities^51^^- ^^55^. In a recent meta-analysis, it was reported that β-alanine supplementation provided ergogenic benefits for high intensity exercise lasting 60 to 240 s^56^.

### Duration of β-alanine Supplementation and Optimal Dosage for Muscle Carnosine Concentration

β-alanine supplementation for 4 to 12 weeks increased muscle carnosine content by 15 to 85%^52^. Stellingwerff et al^57^ examined the effects of two β-alanine dosing protocols on muscle carnosine synthesis. Supplementation involved 3.2 g/d of β-alanine in the first 4-weeks with subsequent intake of 1.6 g/d for the rest of the period; the second group was supplemented daily with 1.6 gram of β-alanine for 8-weeks. Supplementation with 3.2 g/d and 1.6 g/d resulted in about a 2-fold increase of carnosine in tibialis anterior and gastrocnemius muscles compared to 1.6 g/d β-alanine for 8-weeks. The authors suggested that the increase in muscle carnosine depends only on the total amount of β-alanine ingestion and not on the initial muscle carnosine concentration, muscle fibre type or daily amount of β-alanine intake. Recently, Stegen et al^58^ optimized the maintenance dose of β-alanine for elevated muscle carnosine content. In their study, 34 subjects (men and women) were supplemented with 3.2 g/d β-alanine over 46 days and then, divided into half, the participants resumed the β-alanine supplementation for the next 6 weeks with a maintenance dose of 0.4 g/d, 0.8 g/d or 1.2 g/d. Intake of 1.2 g/d β-alanine was demonstrated the optimum dose to maintain muscle carnosine at 30-50% above the baseline. With the intention of achieving high muscle carnosine content, β-alanine can be taken with higher dosages (i.e. 4-6 g/d) for 4-10 weeks^59^. Also, the washout time, i.e. the time of carnosine return to its baseline value, is between 6 to 20 weeks^57^^, ^^60^. Collectively, it appears that 3-6 g/d of β-alanine for a period of 4-10 weeks pre-competition is the appropriate dose. However, to keep high muscle carnosine content, a maintenance dosage of 1.2 g/d seems to be the most effective. Furthermore, it should be mentioned that paresthesia is, as far as we know, the only reported side effect of β-alanine supplementation, but inhibited by slow-release capsules^61^ or by dividing intake into repeated low doses (a separation of the doses every 3–4 h).

### β-alanine Timing

Only a few studies examined the effects of the timing of β-alanine supplementation. Stegen et al^62^ demonstrated that β-alanine intake during meals (containing carbohydrate and protein) enhanced muscle carnosine content compared with in-between meals (64% vs. 41%). However, when a comparison was made with slow-release tablets, no differences were observed in muscle carnosine content. It was speculated that insulin may stimulate β-alanine uptake and augment muscle carnosine loading meditated by Na+/K+ pumps in skeletal muscle cells. However, it is not clear whether only carbohydrate ingestion with β-alanine could augment muscle carnosine content. Further research is warranted to examine co-ingestion of carbohydrate and β-alanine on muscle carnosine content and β-alanine transporters.

### Creatine

First alluded to by the French physicist Michel-Eugène Chevreul in 1835, creatine (i.e. methylguanidoacetic acid) is a non-protein nitrogen compound found mainly in skeletal muscle (95%), but also in heart, brain and other tissues^63^. Creatine has been available for more than 20 years as a dietary supplement. Creatine is a critical part of the phosphagen energy system and involved in ATP regeneration in the creatine kinase reaction. Indeed, initial interest in creatine intake focused on the ability to match cellular ATP production and demand during intense, repeated bouts of exercise. Endogenous biosynthesis of creatine occurs mainly in the liver via the synthesis of arginine, glycine and typically methionine (as S-adenosyl-methionine) at a rate of ~1-2 g/ day^64^. Humans can ingest creatine in beef, pork and certain fish, albeit in small amounts. Under normal circumstances, the rate of endogenous creatine production is approximately equal to its rate of non-enzymatic degradation into creatinine. However, when creatine is supplemented in doses of 3-5 grams or higher for several weeks, creatinine levels will increase as the excess creatine is harmlessly filtered and excreted into the urine.

### Type and Role of Creatine Supplementation

Plasma bioavailability of creatine depends on the intake of the form of creatine, with creatine pyruvate resulting in higher peak creatine concentrations than creatine monohydrate and creatine citrate^65^. However, greater benefits to strength, lean mass and/or performance remain to be demonstrated with these forms of creatine (vs. monohydrate). The long-term safety of creatine monohydrate has been well established while data remain anemic for creatine salts^66^^- ^^68^. During the first few days of creatine supplementation, body weight may increase by several kilograms, an effect attributed to osmotic stimulation of intra- and extra-cellar water retention and secondarily protein and/or glycogen accretion^70^^, ^^69^^, ^^63^. In addition to the aforementioned increase in the capacity of the phosphagen energy system, creatine supplementation has also been shown to serve as a co-factor in gene transcription. In this scenario, oral creatine supplementation increases several myogenic regulatory factors (e.g. myogenic differentiation antigen, myogenin, and muscle regulatory factor 4) that play a role in the differentiation of satellite cells (myoblasts) into myonuclei and eventually mature muscle fibers. Thus, under certain circumstances (i.e. intense exercise and supplementation), creatine appears to increase the myonuclear protein domain^71^. It was also demonstrated that creatine supplementation can enhance glycogen storage in muscle^72^^, ^^73^ secondary to adenosine monophosphate-activated protein kinase phosphorylation, glucose transporter type 4 up regulation, and/or increases in insulin sensitivity. Regardless of the potential mechanisms, creatine supplementation may be of benefit to endurance athletes under certain circumstances (i.e. high carbohydrate intake and intense exercise), although any improvement in endurance performance is likely to be modest at best. In addition to these physical performance effects, 5 g per day of creatine supplementation has also been shown to improve “fluid intelligence” (i.e. the ability to reason quickly and think abstractly) in vegetarians as well as improve certain memory tests in elderly subjects^74^^, ^^75^

### Timing and Dose of Creatine Ingestion

Creatine storage can be augmented by 2 common methods of creatine supplementation including a loading phase with ingestion of 20-25 g creatine (0.3 g/kg day) (almost every 4 hours) for 5-7 days , followed by ~3-5 g (0.03 g/kg day) per day thereafter as a maintenance dose. The creatine loading phase may increase body weight about 2% due to increased muscle water content and the osmotic effect of increased intracellular concentrations of phosphocreatine and creatine to increased muscle glycogen storage^76^, which may impair weight-related performance in running and in other sports in which body weight influenced energy demand77. In line, Sculthorpe et al^78^ reported that 25 g creatine supplementation for 5 days reduced active range of motion of shoulder extension, abduction and ankle dorsiflexion and contributed this affect due to increased retention of water into cells which increase muscle stiffness and neural outflow from the muscle spindles. The ceiling level of total creatine storage (~150–160 mmol/kg dm) is reached by a creatine loading strategy related to urine creatinine excretion by 48-55%^79^, however, few studies have reported that the maintenance dose of creatine supplementation is enough for enhancing of muscle creatine content^80^ and fatigue resistance^81^.

Recently, in the first study on timing of creatine ingestion, Antonio and Ciccone^7^, demonstrated that 5 g creatine monohydrate ingestion post-exercise had greater benefits for body composition (i.e. gains of fat-free mass and loss of fat mass) compared with pre-exercise creatine ingestion during a 4-week resistance training program in young male recreationally bodybuilders. However, this observation was not confirmed by a study in older adults^82^. In addition, Candow et al^83^ observed that 32 weeks of creatine supplementation (0.1 g/kg) in healthy older adults immediately following resistance training lead to greater lean muscle mass compared with immediately before resistance training and resistance training alone. It was speculated that greater lean muscle mass from post exercise creatine supplementation may be due to an increase of skeletal muscle blood flow during resistance training which would result in higher creatine transport and accumulation in exercising muscles. However, in this study creatine during resistance training increased upper and lower body strength compared with resistance training alone with no difference between pre and post-exercise creatine supplementation. More research is needed to address the issue of timing of creatine supplementation with higher dosage on muscle creatine content, body composition and high-intensity exercise performance. Importantly, exercise enhances the effectiveness of creatine storage in skeletal muscle due to an increase of muscle blood flow during exercise76 as does co-ingestion of creatine with either carbohydrate (94 g per 5 g of creatine) or a combination of carbohydrate plus protein (47 g + 50 g per 5 g of creatine, respectively) enhance muscle creatine storage^84^^, ^^85^ via insulin stimulation effect. Finally, it should be emphasized that the incidence of muscle cramps/pulls/ strains, dehydration, or kidney/liver stress stemming from creatine monohydrate use have been overstated as no placebo-controlled, double-blind studies of healthy subjects have ever reported these effects^69^^, ^^86^^, ^^87^.

## Carbohydrate

During exercise, muscle glycogen and blood glucose are important substrates. It is known that by increasing the exercise intensity, carbohydrate (CHO) becomes the main energy substrate and during prolonged exercise, fat oxidation is increased with a decrease of carbohydrate oxidation. However, carbohydrate is always used^88^ and because storage is limited, carbohydrate intake is important for long-duration performance^89^.

### Optimal Dosage and Timing of carbohydrate

The American College of Sports Medicine recommends 3-12 g/kg/day of carbohydrate for low and high intensities exercise, respectively^5^ and there are specific recommendations of carbohydrate intake before, during, and after exercise to maintain glycogen stores and enhance performance^89^. Collectively, timing and optimal dosage of carbohydrate supplementation is divided into 3 categories: before, during and after exercise.

### Carbohydrate Intake before Exercise

In the 3-4 hours before exercise, athletes should intake 200-300 g of carbohydrates (e.g. pasta, rice and/or bread)^90^^, ^^91^. For some individuals, glucose ingestion in the hour before exercise can result in reactive or rebound hypoglycemia during exercise (15-30 min after the onset of exercise)^92^ and to prevent it, an intake of 15 g of carbohydrate (sports drink, gel or bar) immediately before exercise is recommended to prevent hypoglycemia^92^. A carbohydrate intake at least 15 minutes before the exercise can avoid hypoglycemic symptoms when compared with 45 and 75 minutes pre-exercise^93^.

### Carbohydrate Intake during Exercise

In recent review papers, new carbohydrate intake recommendations during exercise were proposed which depends mainly of the exercise duration^94^^, ^^95^.

#### Exercise lasting less than 60 minutes

CHO intake may enhance performance^146^, but there is evidence that it is not necessary to intake large amounts of CHO during exercise lasting less than 60 minutes because in this case, glycogen is not a determinant of performance^96^. However, some studies showed better performance when carbohydrate was ingested for short duration exercise^97^^, ^^98^. Nowadays, it is known receptors in the oral cavity that can activate some cerebral areas associated with reward^99^ and mouth rinse with CHO seems to be able to enhance performance^100^ without using the gastrointestinal tract. There is also evidence that mouth-rinse can improve performance^101^ similar to CHO beverage intake^102^. Studies have shown improvements between 2% and 3% during exercise lasting approximately 1 h and the effects observed are more profound after an overnight fast, but also occurs after a meal^96^. Future work should focus on the effect of CHO mouth rinse in fed conditions, which is more reflective of a real environment of competition.

#### Exercise lasting more than 60 minutes

Consumption of carbohydrate during exercise of long duration enhances the performance in adults^103^. CHO gels and CHO-containing drinks are the main source of CHO during races, whereas the solid form is the least ingested^104^. All the CHO forms can be ingested during exercise, because there is no difference in exogenous CHO oxidation when compared the intake of solid, gel and drink forms^105^^, ^^106^. During competition, optimal CHO beverage concentration seems to be in the range of 5-8%, and athletes should aim to achieve a CHO intake of 60 g/h. The limitation of glucose absorption is 1.0-1.1 g/min and it is possible that when large amounts of glucose are ingested, the absorption is a limiting factor that enhances the chance of gastrointestinal problems^107^^-^^110^. Jeukendrup and McLaughlin^94^ suggested that when exercise last more than 2 hours, there are some benefits in performance ingesting different types of CHO (90 g/h). Combined glucose and fructose intake increases the absorption and fluid delivery because glucose and fructose are absorbed by different transporters^111^. Glucose transport across the brush border occurs by sodium-dependent glucose transporter, whereas fructose is absorbed by glucose transporter type 5^112^. A CHO intake in the form of multiple transportable carbohydrates (glucose plus fructose) can be ingested at a rate of 1.5 g/min^113^ to 1.8 g/min^114^. A solution intake with 1.2 g/min of maltodextrin and 0.6 g/min of fructose showed higher carbohydrate oxidation (approximately 1.5 g/min) than 1.8 g/min of only maltodextrin^114^. However, there is no consensus yet on the glucose: fructose ratio intake to optimize CHO oxidation and consequently performance. Most studies used 2: 1 ratio of glucose: fructose^94^^,^^107^^,^^113^^,^^116^^, ^^117^ but a recent study tested a high intake of CHO (1.8 g/ min) in 10 cyclists during 150 min at 50% peak power followed by an incremental test until exhaustion and compared the proportion of fructose and maltodextrin: 0.6 fructose + 1.2 maltodextrin (0.5 ratio), 0.8 fructose + 1.0 maltodextrin (0.8 ratio) and 1.0 fructose + 0.8 maltodextrin (1.25 ratio) on performance. The authors concluded that a 0.8 ratio was better to enhance performance^118^. However, more studies are required to examine the glucose: fructose ratio intake on performance with different exercises intensities, duration and modalities.

#### Carbohydrate Intake after Exercise

Muscle glycogen is an important substrate during prolonged exercise requiring the need to replenish the storage after exercise. For complete glycogen restoration within 24 hours, it is necessary to provide 8-10 g/kg/day of CHO^107^. Immediately after exercise is recommended an intake of approximately 1.2 g/kg/hour of CHO for maximal muscle glycogen synthesis^115^. In addition, for muscle regeneration, is recommended to intake adequate amounts of protein^119^, however this quantity of CHO plus protein may not be tolerated. It is of practical interest that Beelen et al^120^ suggested that the intake of 0.8 g/kg/h of CHO plus 0.4 g/kg/h of protein presents the same glycogen synthesis than 1.2 g/kg/h CHO alone. These recommendations are important for athletes with high training or competition periods and need to replenish glycogen as fast as possible. For an active person, who has more than 24 h of interval for the next training, glycogen can be replaced during the day and post exercise CHO intake could be ingested in lower amounts. Additionally, combined ingestion of glucose and fructose does not seem to accelerate muscle glycogen resynthesis following exercise in trained cyclists^121^.

## Protein

Protein metabolism during and after exercise is affected by sex, age, intensity, duration, and type of exercise^91^. Strength exercise affects muscle protein turnover which can persist for up 72 h and there is evidences that the timing of intake and the protein source during recovery can regulate the protein synthesis and influence muscle hypertrophy^122^. The current Recommended Dietary Allowance is 0.8 g/kg/day for sedentary adults^119^ and for athletes are needed to intake higher amounts of protein, ranging from 1.2-2.0 g/kg/day^5^ The type of protein and consequently digestibility, amount and type amino acids can influence the protein synthesis^122^. Milk intake showed higher myofibrillar protein synthesis rates than beef during the first 2 hours after resistance exercise, but no difference was observed after 5 hours^124^. Phillips^146^ showed greater muscle protein synthesis after beef intake compared to soy intake. Furthermore, a comparison of milk with soy protein revealed a higher fractional synthesis ratio with milk after exercise^125^ and also promoted higher gain of lean mass^126^. Milk presents 4:1 casein-whey protein. A review^127^ compared the effects of whey, casein, and soy on mixed muscle protein fractional protein rate. Higher protein synthesis was observed with whey protein after exercise^127^ which may be due to whey’s leucine content and rapid absorption. Actually, it has become apparent that leucine is the main amino acid to activate the mTOR pathway^6^ and protein synthesis, however if adequate protein is consumed, the additional leucine does not seems to influence lean mass gain^128^^,^^129^.

### Timing of Protein Intake

The timing of protein intake with respect to an exercise bout seems to be an important. Muscle protein synthesis (MPS) seems to be higher when ingested after resistance exercise compared to before exercise^6^; however, there is no consensus. Tipton et al^131^ showed no difference in MPS with protein intake before or after exercise. A recent meta-analysis showed that the total protein intake during the day is more important than the timing^132^. Although the intake of protein close to the exercise could be important^133^, protein synthesis is elevated after 16-48 hour of resistance exercise and this period can be considered as a “window of opportunity” ^130^^,^^133^. Recently, it was demonstrated that an important moment in the 24 h period for protein intake is before sleep. The effect of 40 g casein plus CHO intake 30 minutes before sleep was examined and the authors concluded that protein ingested immediately before sleep improved whole-body protein balance during post exercise overnight recovery^134^. After this study, the same research group showed that an intake of 27.5 g of protein plus 15 g of CHO before sleep promoted higher muscle mass gain when compared with a non-caloric placebo intake after 12 weeks of resistance exercise program in young individuals^135^. However, it is important to emphasize that the group that received protein supplementation before sleep also increased the total protein intake, therefore, it is not known whether the timing or total protein consumption was the most important. More studies are necessary comparing the timing of protein intake with the same total protein intake between the groups.

### Dose of Protein Intake

The amount of protein necessary to stimulate maximum MPS seems to be an important factor to stimulate lean mass gain. Areta et al^136^ tested the effect of 80 g of whey protein throughout 12 h after resistance exercise ingested in different forms: 8 × 10 g every 1.5 h; 4 × 20 g every 3 h; or 2 × 40 g every 6 h. It was concluded that 20 g of protein consumed every 3 h showed higher protein synthesis . It seems that the ideal dose of protein per meal for adults is 20 g per meal^136^^,^^138^^,^^139^ and an intake higher, for example 40 g, does not increase protein synthesis and promotes higher urea synthesis and amino acid oxidation^139^. Additionally, it is important to remember that when protein is ingested in correct doses, co-ingestion with carbohydrate does not increase protein synthesis^140^^,^^141^. Elderly individuals seems to require higher protein intake, mainly after exercise^139^, due the decrease in the capacity of digestion, postprandial distributionn, muscle amino acid uptake, and anabolic intracellular signalization^123^. For this population, is recommended an intake of 30-40 g of protein per meal and immediately post-exercise^138^^,^^142^. Recently, a new recommendation of protein intake per kilo per meal was proposed^143^. The authors suggested an intake of ~0.25-0.34g/kg/meal for younger and ~0.40 g/kg/meal for older individuals^143^. An important information is that this recommendation was based on six retrospective studies, whereas in five studies whey was used as protein source. Whey contains higher amounts of leucine than other protein sources, therefore the recommendation of protein dose in each meal can be higher when foods that contain less leucine are ingested.

## Conclusions

Several nutritional sports foods and supplements are effective at influencing energy supply, with substantial evidence for carbohydrate and creatine supplements^4^ and for physiological buffering agents such as β-alanine and sodium bicarbonate. This paper reviewed the evidence to determine the optimal time and efficacy doses for sports supplements verified by scientific evidences that report a performance enhancing effect in both situation of laboratory and training settings. These sports supplements included; β-alanine, beetroot juice (nitrate), caffeine, creatine, sodium bicarbonate, carbohydrate and protein (Table 1).

It was concluded that β-alanine should be taken at a dosage of 3-6 g along with each meal containing carbohydrate and protein plus a dose of 1.2 g as a maintenance dose following acute β-alanine supplementation. For nitrate-rich beetroot juice, the recommendation is the consumption of 140 ml (8.4 mmol) containing nitrate, 2-3 h prior to middle distance and endurance exercise. Caffeine should be ingested with a dosage of 3-6 mg/(kg bw), 30- 60 min prior to exercise. Creatine is best supplemented in forms of monohydrate with daily intakes of 3-5 g, or for optimal absorption, 20 g divided into 4 daily intakes of 5 g in combination with carbohydrate and protein. Carbohydrate supplementation before exercise is essential to improve exercise performance. It is suggested that 1-4 g/kg carbohydrate is needed 1-4 h before exercise^144^. In addition, carbohydrate mouth rinse can improve exercise performance (~2-3%) mediated by receptors in the oral cavity and the brain, during exercise lasting less than 60 min. When the exercise duration is more than 60 min, the advice is to ingest 90 g/h of mixed carbohydrates (60 g/ h glucose plus 30 g/h fructose). This is important during prolonged endurance events of 3 hours or more, and, 1.2 g/kg/h carbohydrate is required for glycogen repletion immediately post exercise. Finally, Protein should be ingested in each main meal, immediately post exercise, and also before sleeping with an amount of 20-25 g for stimulating muscle protein synthesis.. However, more research is warranted in the field of supplementation timing and optimal dosage to understand what dosage and time point is most critical for the health and exercise performance of athletes. Finally, it is recommended to examine the repeatability of performance effects of supplement intake by multiple trials^42^.

**Table 1.** Summary of practical guidelines for recommended doses, timing and intake duration of sports supplements.

Supplements	Recommendation doses	Timing	Intake duration
β-alanine	3-6 g	Ingestion of β-alanine with a meal containing carbohydrate and protein	4-10 weeks
Beetroot juice (nitrate)	~ 5-9 mmol	2-2.5 h prior to exercise	1-28 days
Caffeine	~ 3-6 mg / (kg bw)	60-90 min prior to exercise	Na
Creatine	20-25 g (loading dose)	Post-exercise creatine ingestion with beverages	5-7 days
	3-5 g (maintenance dose)	containing carbohydrate and protein	4-12 weeks
Sodium bicarbonate	300-500 mg / (kg bw)	60-180 min prior to exercise	1-3 days
Carbohydrate	300-400 g carbohydrate rich meal	3-4 hr prior to exercise	Na
	Carbohydrate mouth rinsing	During exercise lasting less than 60 min	Na
	30-60 g/h glucose or maltodextrin with 6-8% carbohydrate concentration	During exercise lasting 1-2 hr	Na
	90 g/h glucose or maltodextrin + fructose (2:1) with 8-10% carbohydrate solution	During exercise lasting more than 2.5 h	Na
	1.2 g / (kg bw) or 0.8 g / (kg bw) carbohydrate + 0.4 g / (kg bw) protein	After exercise	Na
Protein	20-25 g for young athletes 40 g for elderly athletes	After exercise	Na
	~0.25-0.30 g / kg each meal for young person ~0.40 g / kg each meal for older person	Meal frequency	Na

**Table 2.** Suggested timing of supplementation intake for potential performance effects.

Supplements	Pre-exercise	During exercise	Post-exercise	Meal time	Before sleep
β-alanine	No	No	No	Yes	No
Beetroot juice (nitrate)	Yes	No	No	Yes	No
Caffeine	Yes	No	No	No	No
Creatine	No	No	Yes	Yes	No
Sodium bicarbonate	Yes	No	No	Yes	No
Carbohydrate	Yes	Yes	Yes	Yes	No
Protein	Yes	No	Yes	Yes	Yes
